# The impact of quinoa flour on some properties of ayran

**DOI:** 10.1002/fsn3.1832

**Published:** 2020-08-26

**Authors:** Yüsra Akkoyun, Seher Arslan

**Affiliations:** ^1^ Engineering Faculty Food Engineering Department Pamukkale University Denizli Turkey

**Keywords:** ayran, fermented dairy products, quinoa, quinoa flour, sensory properties

## Abstract

In this study, some physical, chemical, microbiological, and sensory properties of ayran produced from quinoa flour addition at different ratios (0.1, 0.2, 0.3, and 0.4%, w/v) were investigated. The effect of quinoa addition and storage time on pH, titration acidity, serum separation, L values and microorganism counts were statistically significant (*p* < .05). The counts of *Streptococcus salivarus* subsp. *thermophilus* and *Lactobacillus delbruecki* subps. *bulgaricus* had a wide range between 7.13 and 7.52 log CFU/mL and 3.62 and 3.98 log CFU/mlL At the end of the storage, the general appreciation score of the sample containing 0.2% quinoa flour was found to be higher than the other samples.

## Graphical abstract

The total antioxidant activity of ayran samples was observed between 10.38 (µmol TE/L) and 19.89 (µmol TE/L) during storage period. Addition of quinoa flour up to 0.2% in ayran production had a positive effect on *Lactobacillus delbruecki* subps. *bulgaricus* development. While quinoa flour in ayran production was increased, consistency coefficient of samples increased.

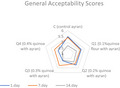

## 1 INTRODUCTION

Yogurt‐style products are the most popular functional products in many countries. In some countries, drinkable fermented dairy products such as doogh, lassi, chaas, and ayran are prepared by adding water in yogurt. Fermented dairy products have many health benefits such as cholesterol lowering, gastrointestinal relief, probiotics, nutrient content, and immune stimulation (Marsh, Hill, Ross, & Cotter, 2014).

Ayran is widely consumed in Turkey and is also beneficial to health. It is preferred because of its nutritional value and desired aroma and taste (Sarhir, Amanpour, & Selli, 2019). Ayran is produced by adding starter culture to standardized milk with adjusted dry matter using water or adding the water in the yogurt (Koksoy & Kilic, 2004).

Quinoa belongs to Chenopodiacea family (Gordillo‐Bastidas, Díaz‐Rizzolo, Roura, Massanés, & Gomis, 2016) and is known as a single‐year, double‐jaw herbaceous plant which is physiologically evaluated in the “C3 plants” group (Dumanoğlu, Işık, & Geren, 2016). It is a gluten‐free product and its motherland is the mountains of the Andes of South America. The most prominent feature of quinoa is a gluten‐free grain. It knows “golden grain” in the Inca language (Navruz‐Varli & Sanlier, 2016). Quinoa is more resistant than some culture plants in adverse environmental conditions. Quinoa can be grown at a height of 4,000 m above sea level and has a high adaptation with its genetic diversity (Jacobsen, 2003). Quinoa has all ten essential amino acids, and protein content of quinoa varies from 12.9% to 16.5%. It is a good source unsaturated fatty acids, dietary fiber vitamins, minerals, and other bioactive compounds (betaine, carotenoids, isoflavones, polyphenols). It contains glucose, fructose, saccharose, and maltose. The starch content in quinoa varies from 58.1% to 64.2%. Quinoa can also be used as different food such as pasta, bread, cookies, and baby food (Gordillo‐Bastidas et al., 2016).

In line with the researches and literature information, it was aimed to obtain a functional food product by adding different amounts of quinoa flour to ayran which is very useful for our health. The effect of quinoa flour on physical, chemical, sensory, and microbiological properties of ayran was also investigated during storage period.

## 2 MATERIALS AND METHODS

### 2.1 Materials

Ayran was produced in the Department of Food Engineering at Pamukkale University, Faculty of Engineering. UHT milk used as material was obtained from the local market of Denizli. Y 811 (10 U)‐coded DVS lyophilized yogurt culture (*Streptococcus salivarus* subsp*. thermophilus* and *Lactobacillus delbrueckii* subsp. *bulgaricus)* was provided by the Maysa Food (İstanbul, Turkey). White quinoa used in the study belonged to A La Çiftçi brand. White quinoa was first shredded in a blender (Waring 8011 ES HGB2WTS3, USA), and then, quinoa flour was passed twice through a laboratory sieve (Retsch, 355 µm pore diameter, Germany). Salt was purchased from local market.

### 2.2 Use of starter culture

Ten units (500 L milk) of Y 811‐coded DVS culture was calculated according to the amount of milk used in production and weighed under aseptic conditions. After heat treatment, starter culture was added to chilled milk at 43°C.

### 2.3 Ayran production

Cow milk was standardized up to 6% dry matter by using water. The heat treatment (90°C–10 min) was applied and milk was cooled to 43 ± 1°C. Starter culture was inoculated at 42°C. Then, divided into 5 groups, the first ayran group (C) did not contain quinoa flour. Experimental ayran Q1, Q2, Q3, and Q4 were prepared from standardized milk added with 0.1%, 0.2% 0.3%, and 0.4% quinoa flour (w/v), respectively. Incubation (4–5 hr, 42°C) was terminated at pH 4.6 ± 0.1, and ayran samples were left at room temperature. Salt (0.75%) was added to ayran. After ayran sample was mixed, they were transferred to 250 ml bottles and kept at refrigerator temperature. The physical, chemical, and microbiological analysis of ayran was done at 1, 7, and 14 days.

### 2.4 Chemical analysis

The fat content and dry matter content were determined by using Gerber method (Bradley et al., 1992) and gravimetric method (Metin & Öztürk, 2002), respectively. The protein content was determined by using Kjeldahl method (AOAC, 1990). The titration acidity was expressed as %lactic acid (Bradley et al., 1992). The pH was measured by using a pH meter (Crison pH‐Meter BASIC 20+, Barcelona, Spain).

### 2.5 Phenolic content and antioxidant activity analysis

The total phenolic content was evaluated by Ertan et al. (2017) with modified procedure. Sodium carbonate (75 g/L) and Folin–Ciocalteu phenolic reagent (1:10, Folin–Ciocalteu phenolic reagent: water) were used during this analysis. 1 ml of ayran samples was placed in the test tube, and 5 mL of FCR and 4 ml of Na_2_CO_3_ were added and stored in the dark for 2 hr. Samples were centrifuged (Nuve 1200 NF, Ankara, Turkey) at 3600 x *g*  for 10 min at 4°C. At the end of the centrifugation, the absorbance of the samples was read at 760 nm on a spectrophotometer (PG Instruments T80 UV/Vis Spectrophotometer, UK). Total phenolic content of the samples was given in mg GAE/L. For antioxidant activity analysis, Thaipong, Boonprakob, Crosby, Cisneros Zevallos, and Byrne (2006) proposed method has been partially modified. Stock solution of DPPH (2,2‐diphenyl‐1‐picrylhydrazyl) was prepared as 24 mg/100 mL methanol and stored at −18°C. Trolox (6‐hydroxy‐2,5,7,8‐tetramethylchroman‐2‐carboxylic acid) solution was used for the calibration curve. For samples or standards, 600 µL was added to 2,400 µL DPPH working solution and allowed to stand in the dark for 1 hr at room temperature. The samples were centrifuged (Nüve NF 1200R, Ankara, Turkey) at 4^○^C at 3600 x *g*  for 10 min. The end of this period, the absorbances of each mixture were read on a spectrophotometer (PG Instruments T80 UV/Vis Spectrophotometer, UK) at 515 nm. Antioxidant activity results were expressed as µmol Trolox equivalent (TE)/L.

### 2.6 Physical analysis

For serum separation, samples were placed in 100‐mL graduated cylinder and stored at 4°C. On the 1st, 7th, and 14th days of the samples, serum separations were measured by looking at the measurements. The results were given as percentages (Tamuçay Özünlü, Koçak, & Aydemir, 2007). Color was measured on yogurts using a HunterLab colorimeter (Hunter MiniScan Xe, Hunter Associates Laboratory, USA) according to the HunterLab scale that is *L* (lightness), a (red/greenness), and *b* (yellow/blueness) (Arslan & Bayrakçı, 2016).

### 2.7 Rheological analysis

Ayran was performed using the SC4‐21 spindle with the Brookfield Viscometer (Model DV‐II + Viscometer, Brookfield Engineering Laboratories, Inc.) device at 4 ± 1°C. Flow behavior indices (*n*) and consistency coefficients (*K*) were determined by using the power law model (*δ* = *K*(*γ*) *n*, *δ*: shear stress (Pa), and *γ*: shear rate (s^−1^) [Gursoy, Yilmaz, Gokce, & Ertan, 2016]).

### 2.8 Fat analysis

The extraction of the samples was made according to the modified Folch method (Folch, Lees, & Stanley, 1957). Fatty acid methyl esters (FAMEs) were prepared according to the IDF standard (Anonymous, 1999). Approximately 0.1 g of the sample extract was transferred into a centrifuge tube and dissolved in 2 mL of hexane. Subsequently, 0.2 mL of a 2 N KOH solution prepared in methanol was added and centrifuged at 2770 x *g* for 5 min. The clear fractions were taken up in vials and made ready for analysis of fatty acid methyl esters by gas chromatography (Agilent 7820B/FID, USA).

A flame‐ionization detector and a Agilent J&W DB‐FATWAX Ultra Inert column (30 m × 0.25 mm i.d., 0.25‐μm film thickness) were used. The injection volume was 1 μL. According to the temperature program, it increased from 50°C in 2 min and from 50°C to 174°C in 14 min. It was then increased by 2°C per minute to 215°C. It was maintained at this temperature for 25 min. Hydrogen was used as the carrier gas. The injector and detector temperatures were 280°C.

### 2.9 Microbiological analysis

M17 (Biolife İtaliana) Agar was used to determine the number of *Streptococcus salivarus* subsp. *thermophilus*. Inoculation was made from the appropriate dilutions using pour plate method. It was then allowed to incubate at 37°C for 48 hr. MRS (De Man‐Rogosa and Sharp agar, Merck) Agar was used to determine the number of *Lactobacillus delbrueckii* subsp *bulgaricus*. Double pour plate was performed, and samples were incubated 37°C for 72 hr. The results were given as log CFU/mL (Peker & Arslan, 2017).

### 2.10 Sensory properties

Ayran sample was evaluated in terms of appearance, color, odor, consistency, taste, and general appreciation by 40 panelists group from Pamukkale University Food Engineering Department. Panelists scored on the sensory form according to the hedonic scale of 1–7 (Altuğ & Elmacı, 2005; Er Gürmeriç, 2008).

### 2.11 Statistical analysis

The results were evaluated statistically by using SPSS program (SPSS package program, Version 20). Analysis of variance (ANOVA) was used for comparison. Duncan test was used in cases where the difference between samples was significant. Statistically differences were determined at *p* < .05 level.

## 3 RESULTS AND DISCUSSION

### 3.1 Chemical composition

The contents of dry matter, protein, fat, and pH value of milk used in yogurt production were 11.08%, 2.90%, 3.00%, and 6.65, respectively. The contents of protein and fat of quinoa flour were 13.13% and 3.76%, respectively.

Table 1 shows some chemical analysis results for the first day of storage of ayran samples. The differences between the samples of ayran on the protein and dry matter contents were statistically significant (*p* < .05). Ayran, according to Turkish Food Codex, was included in the semiskimmed ayran group (0.8%–1.2% milk fat) (Anonymous, 2009). The protein content of ayran samples changed by 2.06%–2.39%. The addition of quinoa flour affected the protein content. Because quinoa is one of the foods rich in protein, the protein content of quinoa flour used in the study was determined as 13.13%.

**Table 1:** Some chemical properties of ayran samples

Chemical properties	C	Q1	Q2	Q3	Q4
Dry matter (%)	6.66 ± 0.04^a^	6.70 ± 0.08^ab^	6.86 ± 0.03^abc^	6.92 ± 0.23^bc^	7.07 ± 0.11^c^
Protein (%)	2.06 ± 0.14^a^	2.15 ± 0.11^a^	2.17 ± 0.05^a^	2.33 ± 0.06^b^	2.39 ± 0.08^b^
Fat (%)	1.22 ± 0.15	1.17 ± 0.05	1.20 ± 0.08	1.20 ± 0.08	1.25 ± 0.12

Note

Differences between ayran samples ^(a,b,c)^ indicated in different lowercase letters at the same storage time (*p* < .05).

C, control sample; Q1: ayran produced by adding 0.1% quinoa flour; Q2: ayran produced by adding 0.2% quinoa flour; Q3: ayran produced by adding 0.3% quinoa flour; and Q4: ayran produced by adding 0.4% quinoa flour.

The pH value and the titration acidity values of samples are shown in Table 2. The differences of samples and storage time on pH and titration acidity values of the samples were statistically significant (*p* < .05). It was found that the pH value decreased gradually in the samples with addition of quinoa flour and the Q4‐coded ayran sample had the lowest pH value compared to the others. This result shows that the use of quinoa flour reduced the pH value in ayran. Gursoy et al. (2016) reported that pH values of ayran samples ranged from 4.53 to 4.10 during storage period.

**Table 2:** Ayran samples pH and titration acidity (as %lactic acid) during storage

Properties	Storage time (day)	C	Q1	Q2	Q3	Q4
pH	1	4.25 ± 0.07^Bb^	4.20 ± 0.04^Cb^	4.19 ± 0.02^Cb^	4.11 ± 0.07^Ba^	4.10 ± 0.05^Ba^
	7	4.14 ± 0.07^ABb^	4.12 ± 0.04^Bab^	4.12 ± 0.01^Bab^	4.06 ± 0.05^ABa^	4.06 ± 0.04^ABa^
	14	4.05 ± 0.10^Aa^	4.03 ± 0.05^Aa^	4.03 ± 0.02^Aa^	3.99 ± 0.08^Aa^	3.99 ± 0.06^Aa^
Titration acidity	1	0.48 ± 0.02^Aa^	0.50 ± 0.02^Aa^	0.51 ± 0.01^Aa^	0.53 ± 0.02^Aab^	0.56 ± 0.02^Ab^
	7	0.51 ± 0.00^Aa^	0.52 ± 0.01^Aa^	0.52 ± 0.02^Aa^	0.54 ± 0.00^Aa^	0.57 ± 0.02^Ab^
	14	0.58 ± 0.02^Ba^	0.58 ± 0.02^Ba^	0.59 ± 0.05^Ba^	0.61 ± 0.03^Ba^	0.62 ± 0.02^Ba^

Note

The differences between storage times shown in different capital letters (^A,B,C^) in the same ayran samples were significant (*p* < .05). Differences between ayran samples (^a,b^) indicated in different lowercase letters at the same storage time (*p* < .05).

Table 2 shows that titratable acidity in ayran samples varied between 0.48% and 0.62%. The difference between the titratable acidity values between the samples and storage period was found to be statistically significant (*p* < .05).

Titratable acidity values of samples increased significantly in the last day of storage compared to the first day. pH values of ayran samples decreased throughout the storage period. Codină, Franciuc, and Mironeasa (2016) investigated the effects of quinoa flour (0%, 0.2%, 0.6%, 1%, 1.4%, and 2%) in yogurt production. As a result of the research, they found that the addition of quinoa flour caused a decrease in pH values. Curti, Vidal, Curti, and Ramón (2017) found that when adding different amounts of quinoa (1%, 3%, and 5%) to yogurts, the pH of yogurts decreased due to storage, and that the yogurt affected the gel structure, and that it was important to affect consumers.

It was found that these studies supported the pH decrease in our study and the addition of quinoa had a significant effect on pH in fermented products. Acidity influences to the serum separation and rheological properties (Gursoy et al., 2016).

### 3.2 Phenolic content and antioxidant activity

The total phenolic content decreased during storage (Table 3). The total phenolic contents of samples at 1 and 14 day of storage were observed between 197.59 and 225.38 mg GAE/L and 100.01 and 105.32 mg GAE/L, respectively. It was determined that the addition of quinoa did not affect the total phenolic content and antioxidant activity. The total antioxidant activity increased at the 7th day and continued to increase at the 14 th day for all samples. This increase was found statistically significant (except Q4‐coded sample).

**Table 3:** Changes of total phenolic content (mg GAE/L) and total antioxidant activity (µmol TE/L) of ayran samples during storage

Properties	Storage period (day)	C	Q1	Q2	Q3	Q4
Phenolic content	1	222.67 ± 16.16^B^	225.38 ± 20.74^B^	197.59 ± 29.35^B^	210.46 ± 23.84^B^	209.53 ± 20.56^B^
	7	108.74 ± 6.55^A^	103.28 ± 9.62^A^	106.96 ± 10.29^A^	102.23 ± 6.87^A^	104.51 ± 6.35^A^
	14	104.34 ± 19.08^A^	105.32 ± 19.16^A^	101.20 ± 16.05^A^	102.16 ± 18.26^A^	100.01 ± 16.95^A^
Total antioxidant activity	1	10.38 ± 2.48^A^	11.13 ± 4.77^A^	11.71 ± 3.19^A^	12.48 ± 2.68^A^	16.00 ± 2.13^A^
	7	15.85 ± 1.49^AB^	15.03 ± 2.32^AB^	14.71 ± 1.72^AB^	15.98 ± 2.76^AB^	17.53 ± 5.16^A^
	14	19.89 ± 6.37^B^	18.26 ± 4.43^B^	16.16 ± 0.44^B^	18.94 ± 5.96^B^	19.25 ± 4.92^A^

Note

The differences between storage times shown in different capital letters ^(A,B)^ in the same ayran samples were significant (*p* < .05).

In a study on the antioxidant capacity of various fermented milk, the antioxidant capacity of ayran was found to be 0.092 mM TE/kg (Najgebauer ‐Lejko & Sady, 2015).

Çelik (2016) reported that the total phenolic content of kefir produced from different proportions of propolis during the storage period. The study showed that the phenolic content of samples ranged from 0.05 to 1.15 mg GAE/g at the beginning of the storage. Also in this study was observed a reduction in total phenolic content during storage. This result supported the decrease in total phenolic content during storage in our study. Lorusso, Coda, Montemurro, and Rizzello (2018) in their study found that the total phenolic content in yogurt‐like drinks contained quinoa flour between 4.00 and 9.60 mmol/kg.

### 3.3 Rheological properties

The values of the consistency coefficient (*k*) of ayran samples and the flow behavior index (*n*) were determined as a result of rheological measurements performed at 4°C on the first day of storage and shown in Table 4. Flow behavior index of ayran samples varied from 0.72 to 0.93. In this study, the flow behavior index of ayran samples was less than 1. It showed non‐Newtonian pseudoplastic flow behavior. Similar results were also reported by Gursoy et al. (2016).

**Table 4:** Rheological properties of samples

Samples	Flow behavior index (*n*)	Consistency coefficient (K, mPa.sn)	Coefficient of determination (*R* ^2^)
C	0.93 ± 0.01	0.09 ± 0.01	0.98 ± 0.01
Q1	0.91 ± 0.01	0.09 ± 0.00	0.99 ± 0.00
Q2	0.87 ± 0.02	0.10 ± 0.01	0.99 ± 0.00
Q3	0.78 ± 0.02	0.13 ± 0.00	0.99 ± 0.02
Q4	0.72 ± 0.02	0.14 ± 0.01	0.98 ± 0.01

**FIGURE 1:**
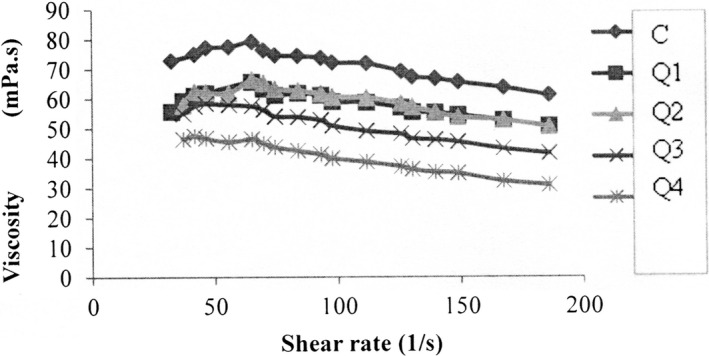
Change of apparent viscosity of ayran samples depending on the shear rate

Apparent viscosity of samples decreased with the severity of shear rate (Figure 1). In the present study, the highest apparent viscosity value was determined in control ayran. These results may be due to the impact of quinoa on casein aggregation.

Because the addition of quinoa flour may cause damage to the gel fragment and a decrease in intermolecular bonding (Codină et al., 2016). Codină et al. (2016) examined the rheological properties of yogurts by adding different amounts of quinoa flour and found that quinoa flour added up to 1% in yogurt production reduced the behavior index and increased the consistency coefficient. They stated that when low level of quinoa flour was added, it can bind water. In addition, researchers explained high level of quinoa flour addition (2%) caused whey loss and damage to the yogurt curd.

In our study, it was found that flow behavior index decreased and consistency coefficient increased with an increase in quinoa flour. In a study examining the rheological properties of ayran using different levels of water and salt, the researchers explained that ayran showed non‐Newtonian behavior based on the power law model (Köksoy & Kılıç, 2003).

### 3.4 Physical properties

The storage period and quinoa addition were a significant factor for serum separation values of ayran samples (*p* < .05). The serum separation value of the control group (C) ranged from 1.50% to 15.75%, while samples containing %0.4 quinoa flour ranged from 5.50% to 26.75% during storage. Sample with 0.4% added quinoa exhibited the highest serum separation values compared with other samples (Table 5).

**Table 5:** Physical properties of ayran during storage time

Physical properties	Storage time (day)	C	Q1	Q2	Q3	Q4
Serum	1	1.50 ± 1.29^Aa^	2.00 ± 1.15^Aa^	4.00 ± 0.00^Ab^	4.00 ± 1.15^Ab^	5.50 ± 1.29^Ab^
	7	12.25 ± 3.20^Ba^	18.50 ± 1.73^Bb^	19.00 ± 1.82^Bb^	20.00 ± 1.82^Bbc^	22.50 ± 0.57^Bc^
	14	15.75 ± 3.77^Ba^	22.50 ± 3.00^Cb^	23.75 ± 0.95^Cbc^	24.75 ± 1.25^Bbc^	26.75 ± 2.06^Cc^
*L*	1	85.48 ± 0.04^Ac^	85.39 ± 0.27^Ac^	85.02 ± 0.31^Abc^	84.79 ± 0.33^Aab^	84.43 ± 0.30^Aa^
	7	85.90 ± 0.18^Ac^	85.61 ± 0.18^Abc^	85.51 ± 0.06^Bb^	85.18 ± 0.17^Aa^	84.98 ± 0.30^Ba^
	14	85.96 ± 0.22^Ad^	85.61 ± 0.05^Ac^	85.39 ± 0.13^Bbc^	85.10 ± 0.25^Aab^	84.88 ± 0.22^ABa^
*a*	1	−2.92 ± 0.14^b^	−2.88 ± 0.03^b^	−2.82 ± 0.11^ab^	−2.80 ± 0.08^ab^	−2.67 ± 0.09^a^
	7	−2.89 ± 0.13^b^	−2.90 ± 0.05^b^	−2.92 ± 0.08^b^	−2.85 ± 0.08^ab^	−2.75 ± 0.09^a^
	14	−2.88 ± 0.13^b^	−2.91 ± 0.03^b^	−2.90 ± 0.02^b^	−2.83 ± 0.01^ab^	−2.75 ± 0.05^a^
*b*	1	7.53 ± 0.29^a^	7.59 ± 0.14^ab^	7.74 ± 0.22^ab^	7.66 ± 0.20^ab^	7.90 ± 0.09^b^
	7	7.55 ± 0.24^ab^	7.49 ± 0.17^a^	7.57 ± 0.08^ab^	7.59 ± 0.15^ab^	7.79 ± 0.13^b^
	14	7.67 ± 0.29^a^	7.59 ± 0.19^a^	7.62 ± 0.19^a^	7.64 ± 0.14^a^	7.85 ± 0.17^a^

Note

The differences between storage times shown in different capital letters ^(A,B,C)^ in the same ayran samples were significant (*p* < .05). Differences between ayran samples ^(a,b,c)^ indicated in different lowercase letters at the same storage time (*p* < .05).

The differences between the formulations of the samples and storage on the L values were found to be statistically significant (*p* < .05). On the 1st and 14th days of storage period, whiteness index (L) value was observed to increase gradually. It was observed that the whiteness index of the samples decreased as the concentration of quinoa used increased.

Ayran samples had negative a value and positive *b* value during storage.

While the differences between the a and b values during storage were not statistically significant (*p* > .05), the differences between the samples were found to be statistically significant (*p* < .05). The lowest a (greenness) values among the samples during storage were determined as (Q4) ayran sample containing the most quinoa flour. While the b value of the control sample was determined as the lowest value at the beginning of storage, the difference between the b values of the samples on the 14th day was not found to be statistically significant (*p* < .05).

Saltoğlu (2014) examined the color values of ayran produced by adding scented black grape pulp, and L, and b values were lower in fruit pulp supplemented ayran samples compared to the control group.

In the study of fermented milk produced by adding vegetable proteins, *L*, *a*, and *b* values were calculated between 85.67–89.02, −1,75‐ (−2.42), and 9.49–11.71 values (Akin & Ozcan, 2017).

### 3.5 Fatty acid composition

The saturated fatty acid content and unsaturated fatty acid content of quinoa flour were 13.00% and 86.97%, respectively. Linoleic acid was the most abundant fatty acid in quinoa flour. Elaidic + oleic acid and palmitic acid were the second and third highest fatty acids. Palmitic acid was the principal saturated fatty acids.

The fatty acid profile of the samples is shown in Table 6. Palmitic acid was the most abundant fatty acid in ayran samples. While 21 kinds of fatty acids were determined in ayran samples (except control), C22: 1n9 fatty acidwas not detected in the control group. The fatty acid profiles of the samples were statistically similar (except some fatty acids). The addition of quinoa flour was very small. Therefore, fatty acid profile may be slightly affected by addition of quinoa flour. C 18:1 (Oleic acid+ elaidic acid) was major unsaturated fatty acid.

**Table 6:** Fatty acid profile (%) of ayran.

Fatty acids	C	Q1	Q2	Q3	Q4
C4	2.00 ± 0.07^a^	2.57 ± 0.04^b^	2.59 ± 0.08^b^	2.38 ± 0.13^b^	2.61 ± 0.07^b^
C6	1.58 ± 0.01^a^	1.83 ± 0.03^b^	1.86 ± 0.03^b^	1.76 ± 0.12^b^	1.92 ± 0.02^b^
C8	1.10 ± 0.00^a^	1.13 ± 0.03^ab^	1.20 ± 0.00^bc^	1.15 ± 0.04^ab^	1.24 ± 0.00^c^
C10	2.59 ± 0.00^a^	2.63 ± 0.08^ab^	2.74 ± 0.02^bc^	2.66 ± 0.05^ab^	2.84 ± 0.02^c^
C11	0.04 ± 0.00	0.04 ± 0.00	0.04 ± 0.00	0.04 ± 0.00	0.04 ± 0.00
C12	3.08 ± 0.00^a^	3.12 ± 0.01^a^	3.16 ± 0.03^a^	3.11 ± 0.04^a^	3.28 ± 0.03^b^
C13	0.10 ± 0.00	0.10 ± 0.00	0.10 ± 0.00	0.10 ± 0.00	0.10 ± 0.00
C14	11.20 ± 0.03^a^	11.17 ± 0.02^a^	11.27 ± 0.13^a^	11.25 ± 0.05^a^	11.72 ± 0.03^b^
C14:1	0.91 ± 0.00^a^	0.93 ± 0.00^a^	0.93 ± 0.01^a^	0.93 ± 0.00^a^	0.96 ± 0.00^b^
C15	1.12 ± 0.00^a^	1.12 ± 0.00^a^	1.13 ± 0.01^a^	1.13 ± 0.00^a^	1.16 ± 0.00^b^
C16	30.50 ± 0.27^ab^	30.00 ± 0.00^a^	30.12 ± 0.15^a^	30.26 ± 0.02^a^	31.09 ± 0.46^b^
C16:1	1.43 ± 0.05	1.41 ± 0.01	1.42 ± 0.08	1.49 ± 0.01	1.47 ± 0.04
C17	0.65 ± 0.00	0.62 ± 0.00	0.64 ± 0.00	0.64 ± 0.01	0.65 ± 0.01
C17:1	0.24 ± 0.01	0.25 ± 0.02	0.27 ± 0.00	0.24 ± 0.00	0.23 ± 0.01
C18	13.51 ± 0.12	13.24 ± 0.06	13.21 ± 0.28	13.40 ± 0.31	13.51 ± 0.74
C18:1*	26.10 ± 0.70	26.04 ± 0.10	25.56 ± 0.73	25.56 ± 0.00	23.28 ± 0.13
C18:2 cis	3.15 ± 0.10	3.09 ± 0.00	3.04 ± 0.10	3.17 ± 0.12	3.17 ± 0.10
C18:3n3	0.27 ± 0.02	0.24 ± 0.00	0.24 ± 0.00	0.27 ± 0.00	0.25 ± 0.00
C20	0.18 ± 0.00^ab^	0.19 ± 0.00^b^	0.18 ± 0.00^ab^	0.18 ± 0.00^ab^	0.17 ± 0.00^a^
C20:3n6	0.18 ± 0.00	0.18 ± 0.00	0.18 ± 0.00	0.18 ± 0.00	0.18 ± 0.00
C22:1n9	–	0.06 ± 0.00	0.07 ± 0.00	0.07 ± 0.00	0.07 ± 0.00
SFA	67.65	67.76	68.24	68.06	70.33
MUFA	28.68	28.69	28.25	28.29	26.01
PUFA	3.60	3.51	3.46	3.62	3.60
P/S	0.05	0.05	0.05	0.05	0.05

Note

Differences between ayran samples ^(a,b,c )^indicated in different lowercase letters at the same storage time (*p* < .05).

* C181tr+C18cis (elaidic + oleic acid).

### 3.6 Microbiological properties

The differences between the treatments and storage period on *Lactobacillus (L.) delbrueckii* subsp. *bulgaricus* counts were found to be statistically significant (*p* < .05). When the amount of quinoa flour in ayran production was increased up to 0.2%, development of *L. delbrueckii* subsp. *bulgaricus* positively influenced. *L. delbrueckii* subsp. *bulgaricus* counts of ayran adding quinoa flour were higher than that of sample without quinoa at the beginning of storage.

**FIGURE 2:**
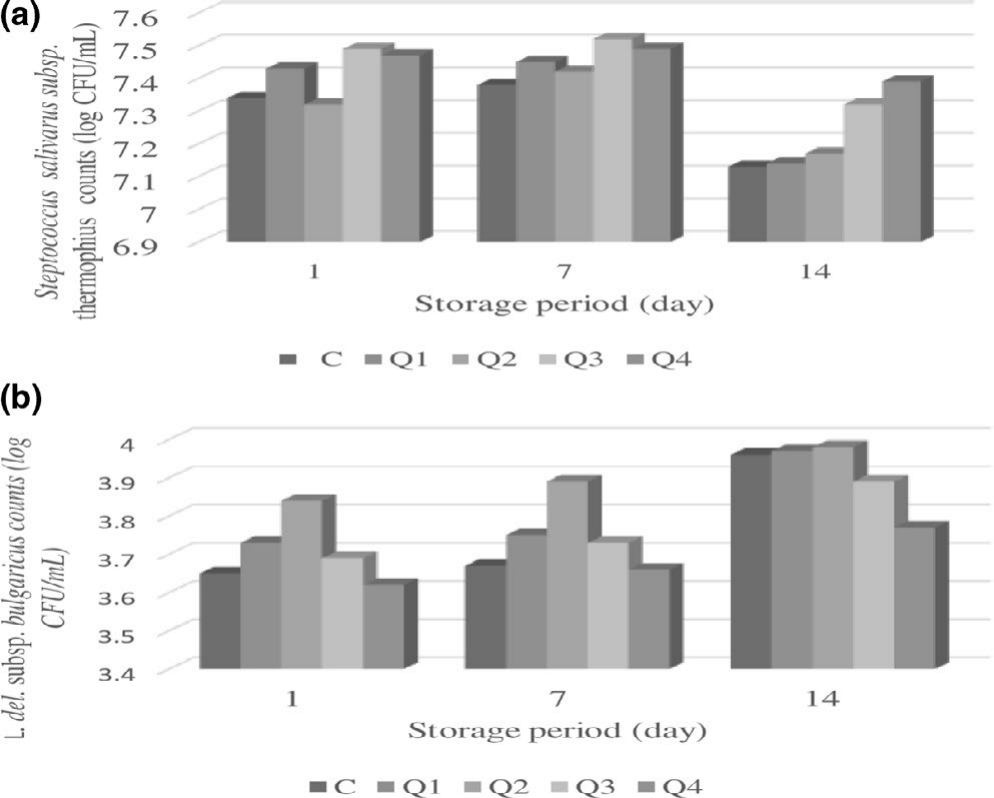
Changes in *S. salivarus* subsp. *thermophilus* (a) and *L. delbruecki* subsp. *bulgaricus* of ayran samples during storage period

*Streptococcus (S.) salivarus* subsp. *thermophilus* count results of ayran samples are shown in Figure 2. Sample formulation and storage period (*p* < .05) had significant effects on *S. salivarus* subsp*. thermophilus* count. In the middle of storage, *S. salivarus* subsp. *thermophilus* count showed a slight increase compared to the first day of storage, and the lowest count results were observed at 15 days of storage.

In a study investigating the effect of quinoa added on fermented milk in different proportions (0, 1, 2, and 3 g/100 g), Casarotti, Carneiro, and Penna (2014) found that quinoa flour did not affect the fermentation time during production but increased acidity during storage. Codină et al. (2016) stated that the addition of quinoa flour had a positive effect on the development of starter yogurt bacteria due to the decrease in pH and increase the total acidity. The results of these studies were compared with the results of our study, and the effect of quinoa flour on microorganism was similar to other studies. The addition of quinoa flour was observed to play an encouraging role in *L. delbrueckii* subsp. *bulgaricus* development.

### 3.7 Sensory properties

The sensory analysis results for the appearance, color, odor, consistency, taste, and general appreciation of ayran samples are shown in Table 7. The data showed that color, odor, taste, and consistency scores were affected by the storage period (*p* < .05). General appreciate score was influenced by the type of sample and storage time.

**Table 7:** Sensory properties of ayran during storage period

Sensory parameters	Storage time (day)	C	Q1	Q2	Q3	Q4
Appearance	1	5.30 ± 0.85	5.15 ± 0.73	5.22 ± 0.89	5.07 ± 1.09	5.27 ± 0.90
	7	5.17 ± 0.95	5.12 ± 0.91	5.20 ± 0.79	5.37 ± 0.83	5.47 ± 0.87
	14	5.07 ± 1.04	5.17 ± 0.87	5.00 ± 0.87	5.05 ± 1.03	5.02 ± 0.94
Color	1	5.15 ± 1.02^A^	5.30 ± 0.88^A^	5.30 ± 0.75^A^	5.10 ± 0.81^AB^	5.22 ± 0.97^A^
	7	5.40 ± 0.81^A^	5.22 ± 0.89^A^	5.40 ± 0.81^A^	5.45 ± 0.67^B^	5.42 ± 0.98^A^
	14	5.25 ± 0.98^A^	5.27 ± 0.78^A^	5.20 ± 0.82^A^	5.02 ± 0.97^A^	5.07 ± 0.94^A^
Odor	1	5.40 ± 0.84^B^	5.30 ± 0.91^A^	5.30 ± 0.82^A^	4.80 ± 1.01^A^	5.12 ± 1.04^A^
	7	5.30 ± 0.88^AB^	5.10 ± 1.00^A^	4.97 ± 0.99^A^	5.10 ± 0.84^A^	5.20 ± 1.18^A^
	14	4.90 ± 1.15^A^	4.87 ± 1.01^A^	4.92 ± 0.99^A^	4.95 ± 1.08^A^	4.77 ± 0.99^A^
Consistency	1	5.05 ± 1.13^AB^	5.25 ± 0.80^B^	5.05 ± 0.98^A^	4.85 ± 1.23^A^	4.85 ± 1.16^AB^
	7	5.35 ± 0.86^B^	5.15 ± 1.07^AB^	4.85 ± 1.05^A^	5.02 ± 0.80^A^	5.30 ± 1.04^B^
	14	4.77 ± 0.97^A^	4.75 ± 0.89^A^	4.82 ± 0.90^A^	4.72 ± 0.87^A^	4.67 ± 0.97^A^
Taste	1	5.27 ± 1.01^B^	4.97 ± 1.02^A^	5.05 ± 1.06^A^	4.45 ± 1.33^A^	4.72 ± 1.26^AB^
	7	5.22 ± 1.02^B^	5.17 ± 1.05^A^	4.90 ± 0.98^A^	5.07 ± 0.97^B^	5.07 ± 1.26^B^
	14	4.47 ± 1.28^A^	4.72 ± 1.10^A^	4.75 ± 0.92^A^	4.55 ± 0.93^A^	4.35 ± 1.21^A^
General appreciation	1	5.47 ± 0.75^Bb^	5.20 ± 0.88^Bab^	5.20 ± 0.82^Aab^	4.75 ± 1.19^Aa^	4.87 ± 1.04^ABa^
	7	5.40 ± 0.95^Ba^	5.12 ± 1.01^ABa^	5.07 ± 0.97^Aa^	5.25 ± 0.80^Ba^	5.20 ± 1.01^Ba^
	14	4.70 ± 1.01^Aa^	4.77 ± 0.80^Aa^	4.87 ± 0.79^Aa^	4.70 ± 0.82^Aa^	4.52 ± 0.84^Aa^

Note

The differences between storage times shown in different capital letters ^(A,B)^ in the same ayran samples were significant (*p* < .05). Differences between ayran samples ^(a,b)^ indicated in different lowercase letters at the same storage time (*p* < .05).

The highest appearance and odor scores were determined in C‐coded ayran sample at the beginning of storage. The Q1‐coded sample was higher of both appearance and color scores than those of other sample at 14 days of storage. Color scores of all samples (except Q1) gradually increased after the first day of storage until the 7th day. Odor and taste scores varied from 4.77 to 5.40 and from 4.35–5.27, respectively. The highest consistency scores were obtained the Q1‐coded sample on the first day of storage and the Q2‐coded sample at the end of storage. It was observed that consistency score decreased below 5 points at the end of storage. A decrease in consistency scores content of ayran may be released of quinoa flour in the structure depending on the time.

The Q2 sample had the highest general appreciate scores (*p* < .05) followed by Q1, C, Q3, and Q4 samples at the end of storage. The general appreciation score was over 5, and general appreciation of all samples was similar at 7 days of storage. The general appreciation of samples decreased at the end of storage.

Carvalho Alves, Corrêa, Pinheiro, and Oliveira (2013) investigated the effect of flour obtained from jaboticaba skin on the sensory properties of yogurt. The researchers evaluated the yogurts on a 9‐point hedonic scale and stated that the sensory properties of yogurt containing 0.1% jaboticaba skin flour ranged between 6 and 7 score (except for color and appearance). Zare, Boye, Orsat, Champagne, and Simpson (2011) found that the lowest sensory score among yogurt samples had the yogurt sample containing 3% lentil flour.

## 4 CONCLUSION

It was found that the pH values of the samples decreased during the storage period and the pH values of samples ranged between 3.99 and 4.25. The addition of quinoa flour reduced the pH of ayran, and the lowest pH value was determined in the Q4‐coded ayran sample containing the highest quinoa flour. The content of protein content and dry matter increased with an increase in the quinoa flour. Different treatment did not cause a significant change in fat content, total phenolic content, and antioxidant activity. Erucic acid was found in samples containing quinoa. Individual effects of different formulations and storage on *L. delbrueckii* subsp. *bulgaricus* and *S. salivarus* subsp*. thermophilus* counts were found statistically significant (*p* < .05). *L. delbrueckii* subsp. *bulgaricus* counts increased during storage period (*p* < .05). *S. salivarus* subsp. *thermophilus* of yogurt drinks slightly increased at the 7th day of storage and then slightly decreased at the 15th day. At the end of storage, 0.2% quinoa flour added sample had the highest general appreciation score.
